# Chemical Characterization, Free Radical Scavenging, and Cellular Antioxidant Properties of the Egadi Island Endemic *Brassica macrocarpa* Guss Leaf Extract

**DOI:** 10.3390/biom14060636

**Published:** 2024-05-29

**Authors:** Adele Cicio, Noemi Aloi, Stefania Sut, Valeria Longo, Francesca Terracina, Stefano Dall’Acqua, Maria Grazia Zizzo, Maurizio Bruno, Vincenzo Ilardi, Paolo Colombo, Claudio Luparello, Rosa Serio

**Affiliations:** 1 Department of Biological, Chemical and Pharmaceutical Sciences and Technologies (STEBICEF), Università degli Studi di Palermo, Viale delle Scienze, 90128 Palermo, Italyfrancesca.terracina@unipa.it (F.T.); maurizio.bruno@unipa.it (M.B.); claudio.luparello@unipa.it (C.L.); rosa.serio@unipa.it (R.S.); 2 Institute for Biomedical Research and Innovation, National Research Council of Italy (IRIB-CNR), Via Ugo la Malfa 153, 90146 Palermo, Italy; noemi.aloi@irib.cnr.it (N.A.); valeria.longo@irib.cnr.it (V.L.); paolo.colombo@irib.cnr.it (P.C.); 3 Department of Pharmaceutical and Pharmacological Sciences, University of Padova, Via F. Marzolo 5, 35131 Padova, Italy; stefania.sut@unipd.it (S.S.); stefano.dallacqua@unipd.it (S.D.); 4 ATeN (Advanced Technologies Network) Center, Viale delle Scienze, University of Palermo, 90128 Palermo, Italy; 5 NBFC—National Biodiversity Future Center, University of Palermo, 90133 Palermo, Italy

**Keywords:** *Brassica macrocarpa*, *Brassicaceae*, antioxidant activity, ROS, scavenging activity

## Abstract

The genus *Brassica* is an important source of food in the Mediterranean diet with documented nutritional and medicinal properties. However, few studies have investigated the phytochemical composition and the biological activity of wild Sicilian taxa. Thus, we aimed to study the chemical profile and the antioxidant potential, in vitro and in LPS-stimulated RAW 264.7 cells, of a methanolic extract of leaves of wild *Brassica macrocarpa* Guss (*B. macrocarpa*) (Egadi Islands; Sicily-Italy). *B. macrocarpa* methanolic extract showed a large amount of glucosinolates and different phenolic compounds. It exhibited antioxidant activity in the DPPH assay and in LPS-stimulated RAW 264.7 cells, being able to reduce NO and ROS levels and NOS2 mRNA expression. Our study demonstrated that Sicilian *B. macrocarpa* methanolic extract, in LPS-stimulated macrophages, efficiently counteracts oxidative stress and displays radical scavenging activity. Future studies are required to identify the contribution of the single phytocomponents, to characterize the action mechanism, and to reveal possible applications in human health.

## 1. Introduction

Consumption of plant-based products in the diet has garnered increasing attention in recent years due to its impact on human health. Evidence suggests a correlation between the intake of plant products and a reduced risk of chronic disorders, including obesity, osteoporosis, cardiovascular, neurodegenerative and inflammatory diseases, and cancer [1,2]. Oxidative stress plays a significant role in the development of these diseases. In the immune system, the balance between oxidants and antioxidants, as well as the generation of reactive oxygen species (ROS), is crucial for immune cell functions, particularly in the induction of cytotoxic activity [3]. Free radicals are generated from enzymatic reactions, such as nitric oxide synthase (NOS), myeloperoxidase (MPO), and hypoxanthine oxidase (HO), or non-enzymatic reactions [4]. Stimulation of immune system cells by pathogenic agents serves as a primary source of these free radicals, eliciting an immune response aimed at eliminating foreign, damaged, or altered cells from the body [4].

Inhibiting oxidative stress is considered an important strategy to mitigate tissue injury during pathological conditions [2,3]. Current medications often come with undesirable side effects [5,6], underscoring the critical need for the discovery of new drugs targeting chronic oxidative stress. While a vast array of plant species holds promise for their potential beneficial effects, rigorous scientific validation is essential for their medicinal use [7,8,9,10].

The beneficial effects of plants are primarily attributed to their richness in bioactive compounds, such as flavonoids, phenolic acids, vitamins, carotenoids, and glucosinolates [11,12,13]. These herbal antioxidants demonstrate remarkable efficacy in scavenging radicals and inhibiting destructive pathways triggered by oxidative stress.

Many of these bioactive compounds, such as glucosinolates and phenolics, are concentrated within the extensive family *Brassicaceae* [14,15]. In particular, wild Mediterranean *Brassicaceae* species are of great interest as sources of nutraceuticals [16,17,18] because the temperate climate and the rich soil of the Mediterranean region enhance the accumulation of phytochemicals [19,20]. Moreover, certain species of wild *Brassica* have served as essential food sources in the traditional diet of Sicilian populations for centuries, and several studies have documented their nutritional and healthy properties when compared to their cultivated counterparts [21,22]. According to The Euro+Med PlantBase [23], the genus *Brassica* includes 11 taxa in Sicily and some of them are endemic and growing in very restricted areas.

Interestingly, to date there are few chemical studies about wild Sicilian *Brassica* focusing on dry seeds, leaves, and aerial parts [17,24,25,26,27].

In this study, we focused our attention on *Brassica macrocarpa* Guss (*B. macrocarpa*). It is a suffrutex plant up to 150 cm high with woody stems, up to 20 mm thick. The leaves are glabrous (15–25 × 10 × 20 cm), ovate, sublyrate, with the apical lobe acute at the margin, and irregularly toothed. The upper leaves gradually become undivided and smaller, whereas the seedling leaves are undivided, ovate, acute, and irregularly toothed. The pedicels (10–20 mm) are erecto-patent with yellowish sepals, whereas the petals are bright yellow. The seeds are reticulate. This species, growing on maritime limestone cliffs and slopes, is an endemic species, with its range limited only to the Egadi Islands (Favignana and Marettimo; Sicily-Italy). The Egadi Islands are known as one of the centers of the diversification of wild taxa within this group [28].

We aimed to investigate the phytochemical profile and beneficial properties of wild Sicilian *B. macrocarpa* during oxidative stress.

In this context, we evaluated the protective effects of *B. macrocarpa* extract on inflammation induced in the murine RAW 264.7 macrophage cell line by lipopolysaccharides (LPS). Macrophages play an important role in the inflammatory response and RAW 264.7 cells represent one of the main in vitro cell culture models for studying inflammation and oxidative stress [29]. LPS is a toxic molecule derived from Gram-negative bacteria cell walls and it is a common inflammatory agent, inducing the release of a large amount of inflammatory and oxidative mediators, which are directly involved in the progression of the inflammatory condition.

To reach our goal, we first performed the phytochemical characterization of our extract from aerial parts of *B. macrocarpa*. Subsequently, we tested the extract’s in vitro antioxidant effect using the DPPH assay. Furthermore, we assessed whether pre-treatment with *B. macrocarpa* extract could efficiently counteract LPS-induced damage in RAW 264.7 cells [29] by improving antioxidant defence and lowering the levels of the main inflammatory markers. Therefore, we evaluated the effects of treatment with *B. macrocarpa* extract in the presence or absence of LPS on intracellular ROS production, as well as on the levels of principal biomarkers related to oxidative stress, including nitrite and NO production.

## 2. Materials and Methods

### 2.1. Plants Materials

The aerial parts of *B. macrocarpa* were collected in February 2022 on Favignana Island, Sicily, Italy. The specimen, identified by Professor Vincenzo Ilardi, was deposited in the STEBICEF Department, University of Palermo, Palermo, Italy (voucher no. 109761).

### 2.2. Extraction of Plant Materials

The collected aerial parts of *B. macrocarpa* were dried at room temperature for 15 days and the dry leaves (76 g) were finely chopped. The plant materials were exhaustively extracted by maceration (1 L × 3 × 72 h) in petroleum ether and successively with methanol as the solvent (1 L × 3 × 72 h). Then, the extracts were filtered through Whatman No. 4 filter paper and the solvent was completely evaporated using a rotary evaporator (Buchi model R-210, Cornaredo, Italy) under reduced pressure (2.5 g petroleum ether extract, 11.0 g methanol extract). The extraction yields were, with respect to dry plant, 3.0 and 11.0%, respectively. The dried residue was stored in an air-sealed analytical container at 4 °C.

### 2.3. Determination of Phytochemicals in Brassica macrocarpa Methanolic Extract by UPLC-MS/MS

#### 2.3.1. Analysis of Glucosinolates

A 10 mg sample of dried extract was dissolved in 2 mL DMSO using an ultrasound bath, then diluted to 10 mL with methanol/water mixture (50%, 20 mL), and the obtained solution was filtered through 0.45 µm filter membrane. A Waters Acquity UPLC equipped with triple quadrupole mass spectrometer coupled to an electrospray ionization source operating in negative ion mode was employed for the quantitative analysis of glucosinolates. A Waters BEH column 2.1 × 100 (1.7 µm) was used and a gradient of 0.1% formic acid in water (A) and acetonitrile (B) was formed, starting from 5% B and reaching 80% B in 4 min, then 95% B in 7 min, and staying isocratic for up to 10 min. Standard solutions of glucosinolates, namely sinigrin, gluconapin, glucoibeverin, glucotropaeonin, glucoerucin, gluconasturcin, glucoraphanin, glucobrassicin, and glucoallyssin (PhytoLab GmbH & Co. KG, Vestenbergsgreuth, Germany), were prepared at concentrations of 5 μg/mL and directly infused in source with the LC flow to optimize the parameters for quantification purposes. The transitions used for the qualitative and quantitative data are summarized in Table 1.

#### 2.3.2. Analysis of Flavonoids

The equipment utilized included an Acquity UPLC equipped with a triple quadrupole mass spectrometer operating in electrospray mode. Additionally, a Varian MS500 ion trap operating in negative mode was employed as a secondary MS detector. Turbo data-dependent scanning was utilized based on the functionality of the instrument, enabling the observation of fragmentation schemes for ion species reaching a selected threshold. The compounds were separated using an Agilent SB C18 3.0 × 100 (1.8 micron) column using a gradient formed by 0.1% formic acid in water (A), acetonitrile (B) and methanol (C). The gradient started at 95% A and 5% B, which was held for 0.5 min, then it reached 92% (A) and 8% (B) in 5 min. Then, it reached 80% (A) and 20% (B) in 15 min, and stayed isocratic for up to 18 min. Then, at 25 min, it reached 50% (A), 40% (B), and 10% (C). At 35 min, it reached 20% (A), 70% (B), and 10% (C), then at 36 min, it reached 0% (A), 85% (B), and 15% (C), and it stayed isocratic for up to 38 min. Then, at 40 min, it reached 100% (B). The compounds were identified by combining the fragmentation of the eluted compounds with the literature data on general flavonoid identification [30] and with specific literature dealing with LC-MS analysis of *Brassica* species [31]. Further confirmation of some of the compounds was finally obtained using reference standards. As reference compounds, kaempferol-3-*O*-glucoside, quercetin-3-*O*-glucoside, kaempferol-7-*O*-glucoside, isorhamnetin-3-*O*-glucoside (PhytoLab GmbH & Co. KG), rutin, and sinapic acid (Sigm-Aldrich, Inc., St. Louis, MO, USA) were used. The transitions used for the qualitative and quantitative data are summarized in Table 2.

### 2.4. DPPH Radical Scavenging Activity

Free radical scavenging activity of the *B. macrocarpa* extract was assessed in vitro by a slightly modified diphenyl-2-picrylhydrazyl (DPPH) assay [32]. The extract, at a concentration range from 7.81 to 1000 μg/mL, was added to 1 mL of absolute ethanol containing 0.1 mM of freshly prepared DPPH. The mixture was shaken vigorously and left to stand for a maximum time of 30 min in the dark at room temperature, and the absorbance was measured using a UV Jasco V760 spectrophotometer at 517 nm.

The DPPH free radical scavenging activity was calculated according to the following equation: DPPH radical scavenging activity (%) = [(Abs of Blank − Abs of Control) − Abs of Sample] (Abs of Blank − Abs of Control).

### 2.5. Cell Culture

The murine macrophage RAW 264.7 cell line was cultured in high-glucose DMEM (Sigma-Aldrich, Inc., St. Louis, MO, USA) supplemented with 10% heat-inactivated fetal bovine serum (Life Technologies, Carlsbad, CA, USA), 100 U/mL penicillin, and 100 µg/mL streptomycin (Sigma-Aldrich, Inc., St. Louis, MO, USA), and maintained in a humidified atmosphere at 37 °C containing 5% CO_2_. After reaching 70–80% confluence, the cells were sub-cultured within two-day intervals. Only cells at passages 6–11 were used for the experiments.

### 2.6. Cell Viability Assay

Cell viability was assessed by the 3, 4, 5-dimethylthiazol-2-yl-2-5-diphenyltetrazolium bromide (MTT) (Tocris, Bio-Techne, Minneapolis, MN, USA) assay. The MTT test measures the conversion of tetrazolium salts to coloured formazan in the presence of metabolic activity. The amount of formazan is proportional to the number of living cells. Raw 264.7 cells (3 × 10^4^ cells/cm^2^) were cultured in 96-well plates and treated with increasing concentrations of *B. macrocarpa* extract (7.81–1000 μg/mL) for 24 h. Untreated cells were used as controls. Subsequently, 100 μL of 0.5 mg/mL MTT dissolved in cell culture medium was added to each well and incubated for 2 h. Then, to dissolve the formed formazan crystals, 100 μL of DMSO was added to each well. A microplate spectrophotometer reader set at 560 nm was used to measure the absorbance of the converted formazan (Synergy HT Microplate Reader (BioTek Instruments, Winooski, VT, USA). The results are presented as percentage of control data.

### 2.7. Nitric Oxide Production

RAW 264.7 cells (5 × 10^5^ cells/well) cultured in 6-well plates were, preliminarily, treated with LPS (*Escherichia coli*, O55:B5 Sigma-Aldrich Inc., St. Louis, MO, USA) at various concentrations (0.1–0.5–1 μg/mL) and for different time (0–3–6–12–24 h) to determine the effective time and concentration at which NO was released without affecting cell viability. Then, cells were treated with increasing concentrations of extract (7.81–1000 μg/mL) for 2 h, and then stimulated with LPS (0.1 μg/mL) for 24 h. The level of NO production induced by LPS stimulation was determined by measuring the nitrite level in the culture media using Griess reagent (Sigma-Aldrich, Inc., St. Louis, MO, USA). Briefly, at the end of treatment, the cells were detached and centrifuged at maximum speed. A 50 μL aliquot of culture supernatant was collected and incubated with Griess reagent, following the manufacturer’s instructions, for 10 min at room temperature. The absorbance was read at 540 nm by a microplate reader (Synergy HT Microplate Reader, BioTek). Untreated cells and cells stimulated with LPS were used as positive and negative controls, respectively. The results are presented as percentage of LPS control data.

### 2.8. Total RNA Extraction and cDNA Synthesis and Real-Time PCR Analyses for NOS2 mRNA Expression

RAW 264.7 macrophages were seeded at a density of 1.25 × 10^5^ cell/well in 24-well tissue culture plates and cultured in high-glucose DMEM medium supplemented with heat inactivated 10% fetal bovine serum (Sigma-Aldrich, Inc., St. Louis, MO, USA) and 1% antibiotic (penicillin 100 U/mL, streptomycin sulphate 100 mg/mL, Invitrogen, San Diego, CA, USA). After 24 h of incubation at 37 °C containing 5% CO_2_, cells were washed in 1X PBS w/o Ca^2+^ and Mg^2+^, treated with increasing concentrations of *B. macrocarpa* extract (from 125 to 1000 μg/mL), and incubated at 37 °C under 5% CO_2_ for 2 h. Subsequently, LPS (0.1 μg/mL) was added, and cells were incubated for a further 24 h at 37 °C under 5% CO_2_. Total RNA was extracted with the PureLink^®^ RNA Mini Kit (Ambion, Life Technologies, Milan, Italy), according to the manufacturer’s protocol. Total RNA was quantified using a Nanodrop One (Thermo Fisher Scientific, Milan, Italy) and 2.5 μg/reaction of RNA template was retro-transcribed using the High-Capacity cDNA Reverse Transcription kit (Applied Biosystems, Thermo Fisher Scientific, Milan, Italy). The cDNA was diluted up to 100 μL in DNAse/RNAse free water and real-time analyses were performed using the Applied Biosystems StepOnePlus™ Real-Time PCR System and Sybr Green technology. Specifically, the amplification reactions were performed using 1–100 ng of cDNA in PowerUp™ SYBR™ Green Master Mix (Applied Biosystems, Thermo Fisher Scientific, Milan, Italy) and 200 nM of specific mouse NOS2 primers in a final volume of 20 µL. The levels of expression of NOS2 were normalized using GAPDH as the housekeeping gene and determined by the 2^−ΔΔCT^ method. The NOS2 and GAPDH primer sequences are reported in Table 3. The PCR cycling conditions included an initial uracil-DNA glycosylase (UDG) activation step at 50 °C for 2 min, followed by DNA polymerase activation at 95 °C for 2 min, 40 cycles of two-step PCR denaturation at 95 °C for 15 s, and annealing/extension at 60 °C for 1 min. The amplification phase was followed by a melt curve stage.

### 2.9. ROS Production

The fluorescent probe dichlorohydrofluorescein diacetate (H_2_DCF-DA), a fluorogenic dye that measures hydroxyl, peroxyl, and other ROS activities within the cell, was used to evaluate ROS levels. After diffusion into the cell, the acetyl groups on H_2_DCF-DA are cleaved by intracellular esterase to yield the nonfluorescent compound, which is rapidly oxidized to highly fluorescent 2′7′ dichlorodihydrofluorescein by ROS. Briefly, RAW 264.7 cells (5 × 10^5^ cells/well) were cultured in 6-well plates, pre-treated with increasing concentrations of *B. macrocarpa* extract for 2 h, and then stimulated with LPS (0.1 μg/mL) for 24 h. Untreated cells and cells stimulated with LPS were used as positive and negative control, respectively. ROS production was evaluated using the ROS Detection Assay Kit (Canvax Biotech, Cordoba, Spain), following the manufacturer’s instructions, and analyzed by flow cytometry. For each analysis, three independent flow cytometric assays were performed on treated and control cells using a FACSCanto instrument (BD Biosciences, Franklin Lakes, NJ, USA) in the FL1 channel (Ex/Em = 485/530 nm). Ten thousand events were assessed, and the obtained data were analysed with the Floreada analysis tool available at https://floreada.io (accessed on 21 July 2023). The mean of fluorescence intensity is reported as percentage of the LPS control group.

### 2.10. Statistical Analysis

All tests were carried out independently in triplicate. Data are expressed as mean ± SEM. All statistical analysis was performed by one-way analysis of variance (ANOVA) followed by Dunnet’s test, when appropriate, using GraphPad (Prism 5.0, Graph-PAD Software, San Diego, CA, USA). A *p*-value < 0.05 was regarded as significant.

## 3. Results

### 3.1. Chemical Composition of B. macrocarpa Extract

**Figure 1:**
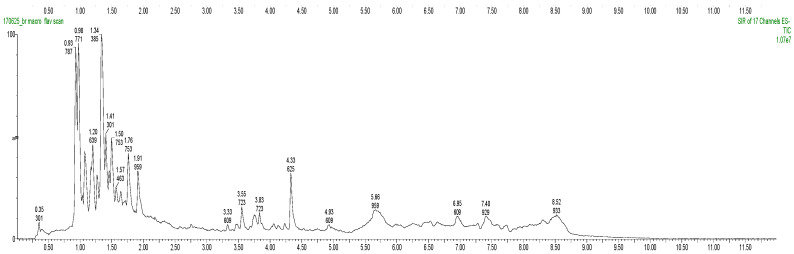
LC-MS chromatogram (BPI) of *B. macrocarpa* methanolic extract.

The LC-DAD-MS^n^ analysis allowed us to identify nine glucosinolates and different phenolic compounds in *B. macrocarpa* extract (Table 4). The negative ion mode was selected because it can efficiently detect both glucosinolates [30,33] and flavonoids [31]. An exemplificative chromatogram is reported in Figure 1.

Glucosinolate identity was confirmed by injection of reference standards, and six derivatives were identified. Glucobrassicin and glucoallyssin were not detectable in the sample. Moreover, several derivatives of phenolic compounds were identified, mostly flavonols. Quercetin and kaempferol derivatives were identified as glycosides presenting different sugar moieties, ranging from one to four units. Rutin, quercetin-3-*O*-glucoside, kaempferol-7-*O*-glucoside, and isorhamnetin-3-*O*-glucoside structures were confirmed by standard comparison. Several isobaric ions sharing the same *m*/*z* were observed, supporting the presence of different glycosidation sites or sugar epimers, as previously described for *B. macrocarpa* [31]. In addition, hydroxycinnamic derivatives were detected and synaptic acid was identified by comparing with reference compounds. A complex pattern of glycosylation was observed for several synapoyl derivatives, and several peaks were ascribed to sinapoyl-feruloyl derivatives. The structures of putative compounds were deduced based on their MS fragmentation, allowing for the identification of the main aglycone moieties [30], and by comparison with the recent literature that considered several *Brassica* species comprising *B. macrocarpa* [31]. Reference standards were then used to confirm the assignments. The list of identified and tentatively identified constituents in the extract is reported in Table 4.

Considering the quantitative data summarized in Table 4, sinigrin and gluconapin were the most abundant glucosinolates. High levels of sinapic acid derivatives were observed, mostly sinapic acid hexoside. Quercetin-3-*O*-dihexoside-7-*O*-hexoside was one of the most abundant phenolic compounds. These data are in good agreement with the report by Picchi et al. [31]. Quantitative data are summarized in the Table 5, showing the amount in mg/g dried weight of extract.

### 3.2. Antioxidant Activity of B. macrocarpa Methanolic Extract

**Figure 2:**
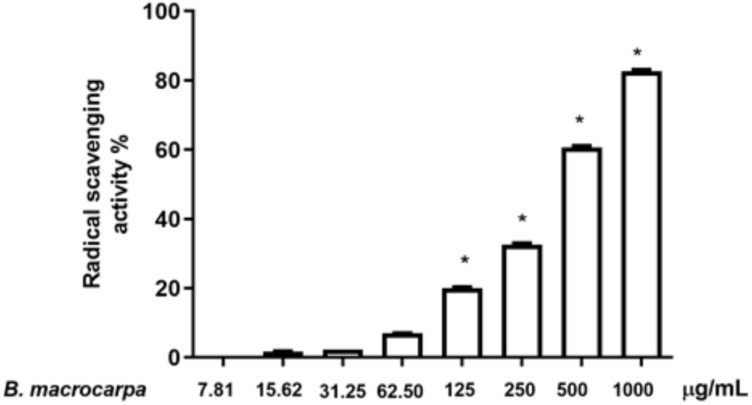
Antioxidant capacity of *B. macrocarpa* methanolic extract measured as DPPH scavenging capacity. Data are expressed as mean ± SEM (*n* = 3). * *p* < 0.05.

The radical scavenging capacity of *B. macrocarpa* methanolic extract was determined by DPPH assay. Antioxidants can transfer either an electron or a hydrogen atom to DPPH to neutralize its free radical character. *Brassica* extract displayed a dose-dependent radical scavenging activity, starting at a dose of 125 μg/mL (Figure 2).

### 3.3. Cytotoxicity of B. macrocarpa Methanolic Extract

**Figure 3:**
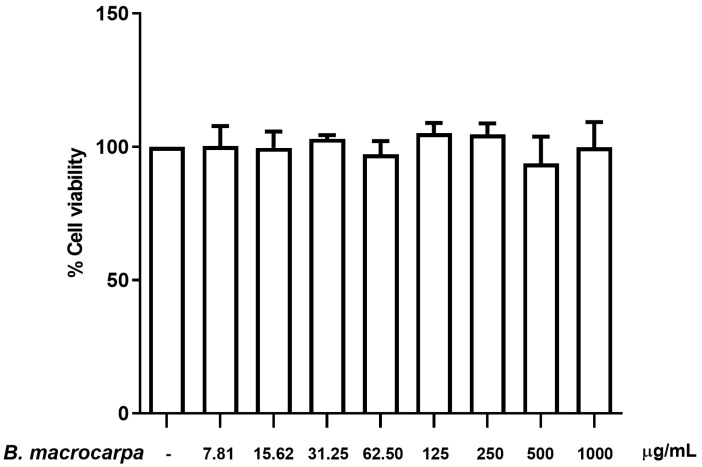
Effect of *B. macrocarpa* methanolic extract on the viability of RAW 264.7 cells. Cells were treated for 24 h with extract at the concentration range from 7.81 to 1000 µg/mL, and cell viability was assessed by MTT assay. Data are mean ± SEM and expressed as the percentage of control cells.

RAW 264.7 cells were exposed to various concentrations of *B. macrocarpa* methanolic extract (from 7.81 to 1000 μg/mL) for 24 h and cell viability was analyzed by MTT assay. *B. macrocarpa* extract did not show any cytotoxic effect at the range of concentrations tested, as demonstrated by the lack of a discernible effect on cell viability (Figure 3). Thus, all of the doses were further used for the experiments.

### 3.4. Effect of the B. macrocarpa Methanolic Extract on Nitric Oxide Production in LPS-Stimulated RAW 264.7 Cells

**Figure 4:**
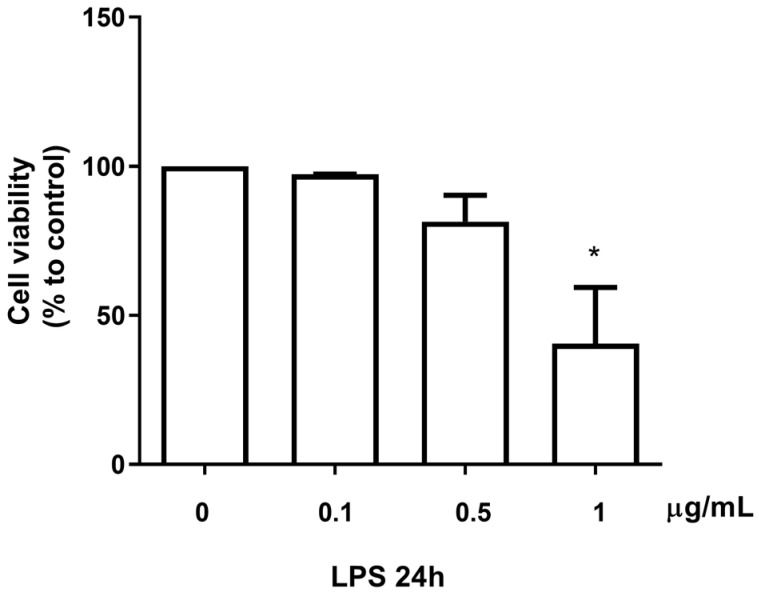
Effect of different doses of LPS on the viability of RAW 264.7 cells. Cells were treated for 24 h with LPS at the concentration range from 0.1 to 1 µg/mL, and cell viability was assessed by MTT assay. Data are mean ± SEM (*n* = 3) and expressed as the percentage of control cells. * *p* < 0.05.

**Figure 5:**
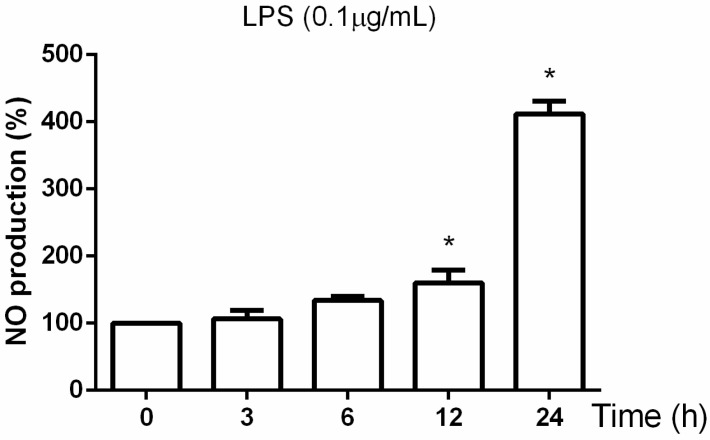
Effects of LPS on nitric oxide production in RAW 264.7 cells at different time points. The amount of nitric oxide produced was determined by Griess assay. Data are expressed as mean ± SEM (*n* = 3). * *p* < 0.05 compared to the untreated cells (time 0).

To determine the level of NO production in LPS-stimulated RAW 264.7 cells, the nitrite released into the culture medium was measured using Griess reagent. Preliminary studies were conducted to find the optimal concentration and exposure time to LPS. As shown in Figure 4 and Figure 5, LPS at a concentration of 0.1 μg/mL for 24 h did not affect cell viability and increased NO production.

**Figure 6:**
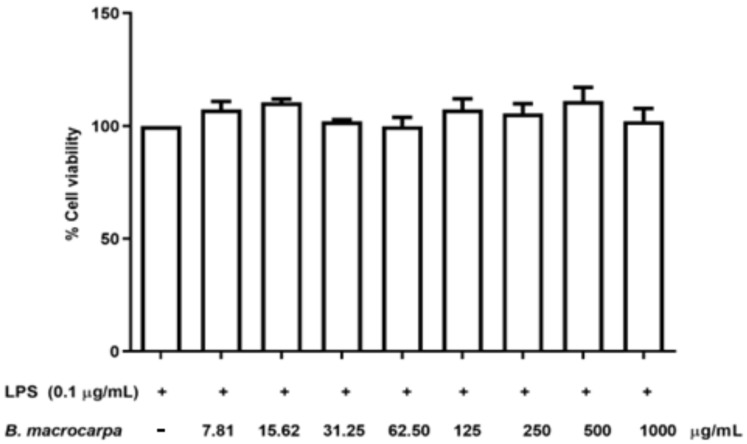
Effect of the joint application of LPS (0.1 μg/mL) and *B. macrocarpa* methanolic extract (concentration range from 7.81 to 1000 µg/mL) on the viability of RAW 264.7 cells. Cell viability was assessed by MTT assay, and no toxicity was observed. Data are mean ± SEM (*n* = 3) and expressed as the percentage of control cells.

Moreover, as indicated in Figure 6, co-treatment with LPS (0.1 μg/mL) and *B. macrocarpa* methanolic extract (7.81 to 1000 μg/mL) did not affect cell viability.

**Figure 7:**
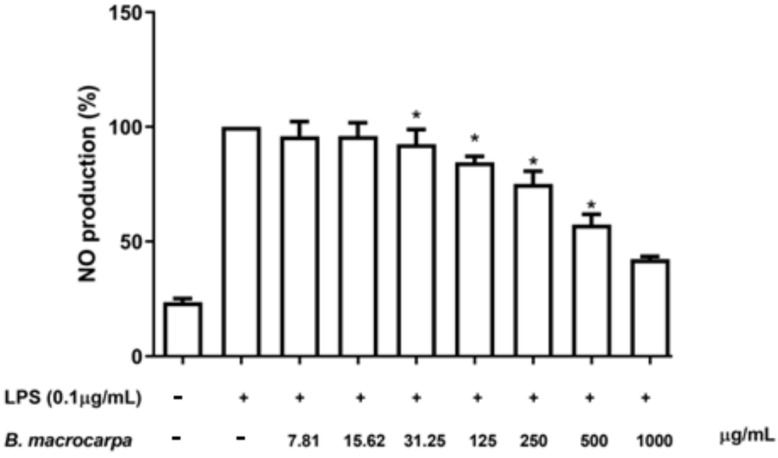
Effects of *B. macrocarpa* methanolic extract on nitric oxide production in LPS-stimulated RAW 264.7 cells. The amount of nitric oxide produced was determined by Griess assay. Data are expressed as mean ± SEM (*n* = 3). * *p* < 0.05 compared to the cells treated with LPS alone (LPS group).

Next, *B. macrocarpa* methanolic extract at the concentration range from 125 µg/mL to 1 mg/mL significantly reduced, in a concentration-dependent manner, NO production levels in LPS-stimulated RAW 264.7 cells (Figure 7).

### 3.5. Effect of the B. macrocarpa Methanolic Extract on NOS2 mRNA Expression in LPS-Stimulated RAW 264.7 Cells

**Figure 8:**
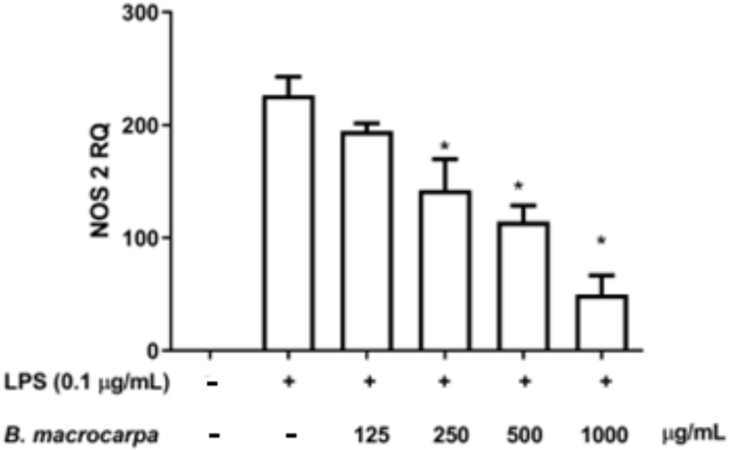
Effects of *B. macrocarpa* methanolic extract on the relative mRNA expression levels of NOS2 in LPS-stimulated RAW 264.7 cells. The cells were treated with the extract for 2 h and then stimulated with LPS (0.1 μg/mL). The mRNA levels were measured by qRT-PCR. Data are expressed as mean ± SEM (*n* = 3). * *p* < 0.05 compared to the cells treated with LPS alone (LPS group).

Because NO production is regulated by the enzyme nitric oxide synthase 2 (NOS2), we investigated the effect of increasing *B. macrocarpa* methanolic extract concentrations on NOS2 expression in LPS-treated RAW 264.7 cells. Indeed, cells incubated in the absence or in the presence of the *B. macrocarpa* extract at the concentrations able to reduce nitrite levels (from 125 µg/mL up to 1000 µg/mL) for 2 h were challenged with LPS (0.1 μg/mL) for 24 h. Total RNA was extracted and the level of expression of the NOS2 gene was analyzed by qRT-PCR. The data obtained showed that NOS2 mRNA expression in unstimulated RAW 264.7 cells was hardly detectable, whereas LPS treatment significantly increased NOS2 expression. The level of NOS2 mRNA was markedly inhibited by pretreatment with *B. macrocarpa* methanolic extract in a concentration-dependent manner (Figure 8).

### 3.6. Effects of B. macrocarpa Methanolic Extract on ROS Production in RAW 264.7 Cells

**Figure 9:**
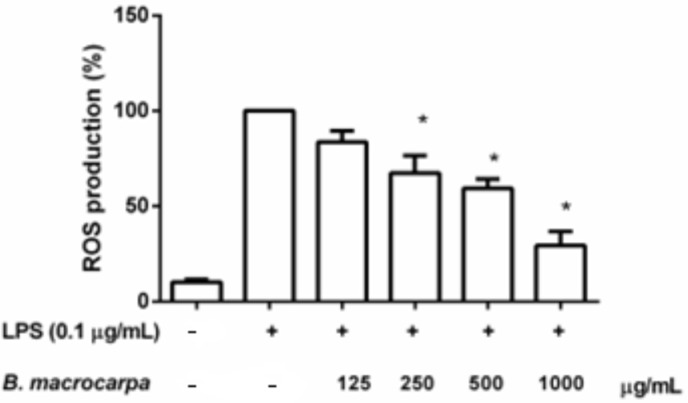
Effects of *B. macrocarpa* methanolic extract on ROS production in LPS-stimulated RAW 264.7 cells. The amount of ROS produced was determined using the H_2_DCF-DA assay. Data are expressed as mean ± SEM (*n* = 3). * *p* < 0.05 compared to the cells treated with LPS alone (LPS group).

Due to the encouraging effect of *B. macrocarpa* methanolic extract on nitrite levels, we further explored its potential antioxidant properties, examining the effect of *B. macrocarpa* extract on reactive oxygen species (ROS) production in RAW 264.7 macrophages stimulated with LPS. As expected, in macrophages exposed to 0.1 μg/mL of LPS for 24 h, we observed enhanced ROS production when compared to the control (Figure 9). Treatment with *B. macrocarpa* extract at the concentrations able to reduce nitrite levels (125–1000 µg/mL) downregulated ROS production, showing about a 71% decrease at the highest concentration tested (1000 µg/mL). These findings indicated that *B. macrocarpa* methanolic extract induced antioxidant effects also in the cell model system.

## 4. Discussion

Plants belonging to the family *Brassicacea* are known as one of the important sources of food and medicines worldwide due their high content of dietary antioxidant compounds, including polyphenolics. Indeed, their antioxidant potential has been demonstrated through in vivo and in vitro assays [12,34]. Particularly noteworthy is their ability to scavenge radicals and chelate transition metals. Compounds such as phenolics or glucosinolates contribute to this high antioxidant capacity. These compounds work in a variety of ways to protect cells from oxidative damage, such as preventing reactive oxygen species formation, scavenging radicals, or restoring target molecule damage [12,15].

In our study, we focused on *B. macrocarpa*, a wild *Brassica* species native to Sicily collected on one of the Egadi Islands, Favignana. Wild species have a great deal of potential as sources of bioactive compounds because they strengthen their own chemical defenses by increasing the synthesis of specialized secondary metabolites, such as antioxidants, in order to adapt to challenging environments [35]. Thus, we investigated whether the unique habitat could affect the phytochemical contents and beneficial activities of *B. macrocarpa*.

In our extract, several compounds were detected, which were already described and identified in different *Brassica* spp. [24,26,31,36], although with great variability in their qualitative and quantitative composition.

Moreover, regarding the flavonoids, quercetin and kaempferol glycosides and hydroxycinnamic acids esters were identified, consistent with findings of Picchi et al. [31], which characterized the leaf extract of *B. macrocarpa* from seeds collected on the Egadi Islands and grown on an experimental farm. However, in our extract of *B. macrocarpa*, compared to the previous study, we detected more quercetin derivatives compared to kaempferol derivatives, whilst gentiobiosides were not detected, suggesting that different environmental growing conditions and/or extraction processes can affect phytochemical composition. However, glycosylation of flavonoids strongly enhances their water solubility and thus increases their bioavailability, although antioxidant and most biological activities are usually less pronounced [37].

Moreover, we also analyzed and characterized the glucosinolates, showing a high content in sinigrin. This finding is in agreement with previous research [38] reporting high glucosinolate content in *B. macrocarpa* leaves, with the sinigrin content being about 90%.

Indeed, sinigrin has attracted considerable interest based on its antioxidant and anti-inflammatory activities. Sinigrin is a major glucosinolate associated with the family of glucosides present in members of the family *Brassicaceae*, such as the seeds of black mustard (*Brassica nigra*), Brussels sprouts, and broccoli. This glucosinolate can induce different biological effects and also plays pivotal role in the prevention of DNA damage caused by carcinogens [39]. Sinigrin can also reduce the level of plasma triglycerides, and its breakdown product allyl isothiocyanate is able to suppress nitric oxide production and the stimulation of inducible nitric oxide synthase in LPS-activated J774.1 macrophages [40,41]. *In vivo*, sinigrin administration in LPS-treated rats significantly reduced the urinary levels of nitrate and nitrite, an index of NO production. It was also revealed that sinigrin has antioxidative properties and lowers the level of reactive nitrogen species [42].

Although our study did not investigate the relationship between phytochemicals and biological effects, quantitative analysis of the composition of *B. macrocarpa* extract indicated that glucosinolates and, in particular, sinigrin, are likely the compounds in our extract with potential beneficial effects against oxidative stress. However, further studies are needed to analyze the role played by each compound individually and in combination to elucidate possible interactions between compounds. For example, a recent study by Fusari et al. [43] demonstrated a correlation between antioxidant capacity and many components of different widely consumed *Brassicaceae* species, suggesting a contribution of both sulfur and phenolic compounds to antioxidant effects. Additionally, Fusari et al. [43] indicated that the hydrogen transfer mechanism was the main antioxidant mechanism involved for cruciferous phenolic compounds, while the electron transfer mechanism was predominant for sulfur compounds.

Our findings clearly demonstrate that *B. macrocarpa* extract reduced the stress induced by LPS in macrophages, decreasing the levels of NO and ROS.

Oxidative stress is implicated in numerous noncommunicable diseases, including aging, chronic fatigue, allergic dermatitis, cancer, inflammation, arteriosclerosis, heart and cardiovascular disease, and kidney illness.

Using in vitro assay such as DPPH, a quick, simple, reproducible, and cost-effective method frequently utilized for evaluating the antioxidant potential of drugs, we demonstrated in vitro the scavenging activity of *B. macrocarpa* extract. The ability of our *Brassica* extract to scavenge radicals increased in a concentration-dependent manner, starting at a dose of 125 μg/mL. This radical scavenging activity may be attributed, once more, to the presence of certain compounds, such as glucosinolates, as the correlation between antioxidant activity and the content of these compounds has been reported elsewhere [44,45,46].

The next step was to investigate the antioxidant ability also in a cellular model. Preliminarily, we evaluated possible side effects by testing the cellular toxicity of various concentrations of the extract on murine macrophages over a 24 h period. Our results indicated that the *Brassica* extract is safe, as no cytotoxicity was observed at any of the tested concentrations.

LPS-induced activation of RAW 264.7 macrophages is a common model for screening antioxidative drugs. Indeed, when macrophages are over-activated by inflammatory stimulants, such as the Gram-negative bacterial endotoxin LPS, it is possible to observe high levels of NOS2 expression and an increase in the production of various mediators, such as NO and ROS [29].

NO is easily converted to a stable end-product, nitrite, and then to nitrate. Treatment of RAW 264.7 cells with the extract prior to LPS stimulation demonstrated significant inhibition of nitrite levels, indicating the potential of our extract to alleviate oxidative stress. Since NO is a multifunctional signaling molecule, as has been shown in numerous cell types, the effect of the extract on NO production may have additional impacts on signaling pathways [47]. The *Brassica* extract demonstrated potent radical scavenging activity, suggesting that the reduction of NO production may occur via scavenging of nitrogen radicals. A second possible mechanism may be the reduction of NOS activity. Our results clearly demonstrated that *B. macrocarpa* extract reduced NO production via transcriptional suppression of the NOS2 gene.

ROS are known as signaling molecules associated with host–defense response [48]. They play essential roles in physiological functions. ROS, however, may also contribute to the evolution of inflammatory disorders by functioning as inflammatory mediators. Excessive production of ROS and related species disrupts cellular homeostasis, structures, and functions, leading to oxidative stress [49]. Several studies have linked the consumption of plant foods, which are abundant in antioxidants, to a lower risk of diseases caused by reactive oxygen species [50]. In addition, plant secondary metabolites have also been shown to be able to reduce ROS levels, playing an important role in oxidative stress [51]. Therefore, antioxidants play a pivotal role in maintaining healthy physiological conditions by scavenging ROS linked to the inflammatory response and oxidative stress [4,52,53,54].

Our data indicated a dose-dependent inhibition of ROS generation by *B. macrocarpa* extract. This finding, along with the evidence of the extract’s ability to scavenge free radicals, as suggested by the DPPH test, confirmed its antioxidant action in LPS-stimulated RAW 264.7 macrophages.

ROS and NO activate several biological pathways, such as nuclear factor-kappa B (NF-κB) and mitogen-activated protein kinase (MAPK) signaling pathways [55,56,57], leading to overproduction of these mediators and cytokines, in turn provoking detrimental effects on the etiology of several disorders [55,58]. Thus, one possible treatment strategy to slow the evolution of inflammatory and oxidative disorders is to reduce the generation of inflammatory factors by inhibiting macrophage activation [59,60]. Thereafter, further investigations could be useful to evaluate the effect of *B. macrocarpa* extract on the inflammatory response and to better characterize the action mechanism of the observed preventive antioxidant effect against LPS-induced NO and ROS generation.

## 5. Conclusions

Pre-treatment with *Brassica macrocarpa* extract efficiently counteracted the oxidative stress induced by LPS in RAW 264.7 cells, lowering the main oxidative biomarkers such as ROS and NO, likely via the inhibition of oxidative stress generation and radical scavenging activity. However, further studies are needed to elucidate the specific contributions of specific phytocomponents to these beneficial effects, to clarify the underlying action mechanisms, and to validate the antioxidant activity of the extract *in vivo*. Furthermore, this research holds the potential to strengthen the case for incorporating *B. macrocarpa* extract as a supplement to treat conditions characterized by oxidative stress.

## Figures and Tables

**Table 1:** Main parameters of the standard solutions of glucosinolates used for the qualitative and quantitative analyses.

Compound	[M − H]^−^ *m*/*z*	Fragment Ion	Dwell Time	Cone	Collision Energy
sinigrin	358.0957	96.8426	0.050	36	16
gluconapin	372.0432	96.755	0.050	46	25
glucoibeverin	406.0306	69.8426	0.050	40	20
glucotropaeonin	408.0853	96.9037	0.050	38	16
glucoerucin	420.0853	96.9033	0.050	44	16
gluconasturtiin/iberin	422.0853	96.7476	0.050	46	22
glucoraphanin	436.0411	96.755	0.050	48	22
glucobrassicin	447.0865	96.7555	0.050	46	22
glucoalyssin	450.0555	96.755	0.050	48	22

**Table 2:** Main parameters of the standard solutions of flavonoids used for the qualitative and quantitative analyses.

Compound	[M − H]^−^ *m*/*z*	Fragment Ion	Dwell Time	Cone	Collision Energy
kaempferol-3-*O*-glucoside	447.092	285	0.050	20	20
quercetin-3-*O*-glucoside	463.087	301	0.050	22	20
kaempferol-7-*O*-glucoside	447.092	285	0.050	20	20
isorhamnetin-3-*O*-glucoside	477.1072	299	0.050	35	25
rutin	609.1520	301	0.050	18	16
Sinapic acid	223.063	208	0.050	10	20

**Table 3:** Primer sequences.

Gene	Primer Forward	Primer Reverse
*GAPDH*	5′-GGCCTTCCGTGTTCCTAC-3′	5′-TGTCATCATATCTGGCAGGTT-3′
*NOS2*	5′-CAGGAGGAGAGAGATCCGATTTA-3′	5′-GCATTAGCATGGAAGCAAAGA-3′

**Table 4:** Tentative identification of the major secondary metabolites in *B. macrocarpa* extract and parent ions of the identified compounds (*m*/*z*). Putative compounds were identified on the basis of MS data and the literature [31], as well as by standard comparison.

Glucosinolates	[M − H]^−^
Sinigrin *	358.0957
Gluconapin *	372.0432
Glucoibeverin *	406.0306
Glucotropaeonin *	408.0853
Glucoerucin *	420.0853
Gluconasturtiin */Iberin	422.0853
Glucoraphanin *	436.0411
Glucobrassicin *	447.0865
Glucoalyssin *	450.0555
**Phenolics and flavonoids**	[M − H]^−^ and fragments
Quercetin-3-*O*-hexoside-7-*O*-hexoside	625.13, 301.1
Quercetin-3-*O*-hexoside-7-*O*-hexoside	625.13, 301.2
Quercetin-3-*O*-hexoside-7-*O*-hexoside	625.13, 301.2
Quercetin-3-*O*-dihexoside-7-*O*-hexoside	787.13, 601.0, 301.1
Quercetin-3-*O*-dihexoside-7-*O*-hexoside	787.13, 601.1, 447.1, 301.2
Quercetin-3-*O*-trihexoside-7-*O*-hexoside	949.23, 447.2, 301.1
Quercetin-3-*O*-trihexoside-7-*O*-hexoside	949.23, 447.1, 301.1
Kaempferol-3-*O*-glucoside *	447.2, 285.2
Kaempferol-3-*O*-dihexoside-7-*O*-hexoside	771.2, 285.1
Kaempferol-3-*O*-dihexoside-7-*O*-hexoside	771.2, 285.1
Kaempferol-3-*O*-trihexoside-7-*O*-hexoside	933.25, 285.1
Kaempferol-3-*O*-trihexoside-7-*O*-hexoside	933.25, 285.1
Isorhamnetin-dihexoside	639.15, 299.1
Rutin *	609.14, 301.1
Quercetin-3-*O*-glucoside *	463.09, 301.1
Kaempferol-7-*O*-glucoside *	447.09, 315.1
Isorhamnetin-3-*O*-glucoside *	477.11, 315.1
Sinapic acid *	223.06
Sinapic acid hexoside	385.11, 223.1
Sinapic acid hexoside	385.11, 223.1
Sinapic acid hexoside	385.11, 223.2
Disinapoyl-gentiobioside	753.2, 529.1
Disinapoyl-gentiobioside	753.2, 529.1
Sinapoyl-feruloyldiglucoside	723.21, 223.1
Trisinapoyl-diglucoside	959.28, 223.1
Disinapoyl-feruloyldiflucoside	929.27

* Confirmed by comparison with authentic standard.

**Table 5:** Amount (mg/g) in the *B. macrocarpa* extract of identified secondary metabolites.

Glucosinolates	mg/g Dried Weight
Sinigrin *	92.80
Gluconapin *	17.26
Glucoibeverin *	0.06
Glucotropaeonin *	0.17
Glucoerucin *	0.06
Gluconasturtiin */Iberin	0.05
Glucoraphanin *	0.03
**Phenolics and flavonoids**	mg/g
Quercetin-3-*O*-hexoside-7-*O*-hexoside	15.30
Quercetin-3-*O*-hexoside-7-*O*-hexoside	15.65
Quercetin-3-*O*-hexoside-7-*O*-hexoside	18.46
Quercetin-3-*O*-dihexoside-7-*O*-hexoside	30.65
Quercetin-3-*O*-dihexoside-7-*O*-hexoside	5.52
Quercetin-3-*O*-trihexoside-7-*O*-hexoside	19.05
Quercetin-3-*O*-trihexoside-7-*O*-hexoside	7.30
Kaempferol-3-*O*-glucoside *	0.36
Kaempferol-3-*O*-dihexoside-7-*O*-hexoside	29.22
Kaempferol-3-*O*-dihexoside-7-*O*-hexoside	8.39
Kaempferol-3-*O*-trihexoside-7-*O*-hexoside	18.12
Kaempferol-3-*O*-trihexoside-7-*O*-hexoside	8.11
Isorhamnetin-dihexoside	16.57
Rutin *	12.83
Quercetin-3-*O*-glucoside *	4.59
Kaempferol-7-*O*-glucoside *	3.83
Isorhamnetin-3-*O*-glucoside *	3.34
Sinapic acid *	2.34
Sinapic acid hexoside	2.97
Sinapic acid hexoside	40.82
Sinapic acid hexoside	6.82
Disinapoyl-gentiobioside	2.02
Disinapoyl-gentiobioside	1.75
Sinapoyl-feruloyldiglucoside	7.02
Trisinapoyl-diglucoside	13.28
Disinapoyl-feruloyldiflucoside	2.11

* Confirmed by comparison with authentic standard.

## Data Availability

Data will be made available from the corresponding author upon reasonable request.
